# Usefulness and importance of echocardiography in the diagnosis of pediatric pulmonary hypertension

**DOI:** 10.1186/s44348-024-00022-5

**Published:** 2024-10-08

**Authors:** Hee Joung Choi

**Affiliations:** Department of Pediatrics, Keimyung University Dongsan Hospital, Keimyung University School of Medicine, Daegu, Republic of Korea

Pediatric pulmonary hypertension (PH) is a progressive condition that has a high risk of morbidity and mortality. Unlike adults, the frequency of idiopathic pulmonary artery hypertension (PAH) is low and there is a possibility of reversal with treatment of the underlying disease, so accurate diagnosis and prompt treatment are important in children [1]. Various methods are used to diagnose PH, but it is not feasible to perform complete evaluations in children [2].

Right heart catheterization is the gold standard for the definitive diagnosis of PAH [3], but it is invasive and can lead to several complications [1, 3]. In particular, for young children, the incidence of catheterization complications including death is as high as 5.9%, and sedation is required if cooperation is not possible [1, 4]. Compared to right heart catheterization, echocardiography is noninvasive and can be easily used without sedation; it also involves no radiation exposure [1, 3]. For these reasons, echocardiography is recommended to evaluate pediatric patients suspected of having PH before cardiac catheterization [4].

The diagnostic criteria of PH for children are the same as for adults. Pediatric PH is defined as a mean pulmonary arterial pressure of more than 20 mmHg in children after 3 months of age [3, 5]. In pediatric PH associated with congenital heart disease, PAH is defined as pulmonary capillary wedge pressure less than 15 mmHg as well as indexed pulmonary vascular resistance greater than 3 Woods units × m^2^ [3]. However, there are two considerations in applying this criterion to the diagnosis of pediatric PH using echocardiography. First, because the above criteria are based on resting cardiac catheterization data, it is questionable whether the values measured by echocardiography can be used as is. Second, since the above standards are based on a survey of adults, the question is whether they can be applied to children as well.

The current study by Malakan Rad E et al. [6] addresses these questions. The authors developed a new formula for diagnosing PH using tricuspid and pulmonary valve regurgitation velocities and inferior vena cava diameter on echocardiography, and that formula demonstrated a strong correlation with invasively measured values in children. Additionally, the cutoff values of 32.9 mmHg systolic, 15.0 mmHg diastolic, and 20.7 mmHg mean pulmonary arterial pressure suggested by the new formula closely match the currently used standards for adults. Based on the results of this paper, it seems that the previously presented adult standards from cardiac catheterization can be used to diagnose PH in children based on echocardiography.

Even before measuring pulmonary artery pressure, PAH can be suspected based on the flattening of the interventricular septum with a D-shaped left ventricle or right-to-left cardiovascular shunting [4, 7]. These dichotomous variables, along with pulmonary artery pressure measurements, are also frequently used for PH diagnosis through echocardiography. In the pediatric group, right ventricle longitudinal systolic function by tricuspid annular plane systolic excursion and pulmonary artery acceleration time determination is useful in children with suspected or confirmed PH [4]. However, Elaheh Malakan Rad et al. [6] reported the low accuracy of dichotomous variables in children. This is because time variables such as pulmonary artery acceleration time depend on heart rate, and age-specific standards that take into account the high heart rate of children are needed [8, 9].

Despite several limitations, echocardiographic evaluations with a multiparametric approach in pediatric PH are a valuable noninvasive tool in screening, diagnosing, and assessing pediatric PH. Further research will be needed to overcome the limited data on normal hemodynamic values in pediatric populations and to determine optimal cutoff standards for echocardiography. 

## Data Availability

Not applicable.

## Abbreviations

PAH: Pulmonary artery hypertension
PH: Pulmonary hypertension
